# Supplementary material for: Modulating p38 MAPK signaling by proteostasis mechanisms supports tissue integrity during growth and aging
Source: Nat Commun. 2023 Jul 28;14:4543. doi: 10.1038/s41467-023-40317-7 (PMC10382525; doi:10.1038/s41467-023-40317-7)
Supplement: Supplementary file 1 — Supplementary Information [file 41467_2023_40317_MOESM1_ESM.pdf]

Supplementary Figures 1-9

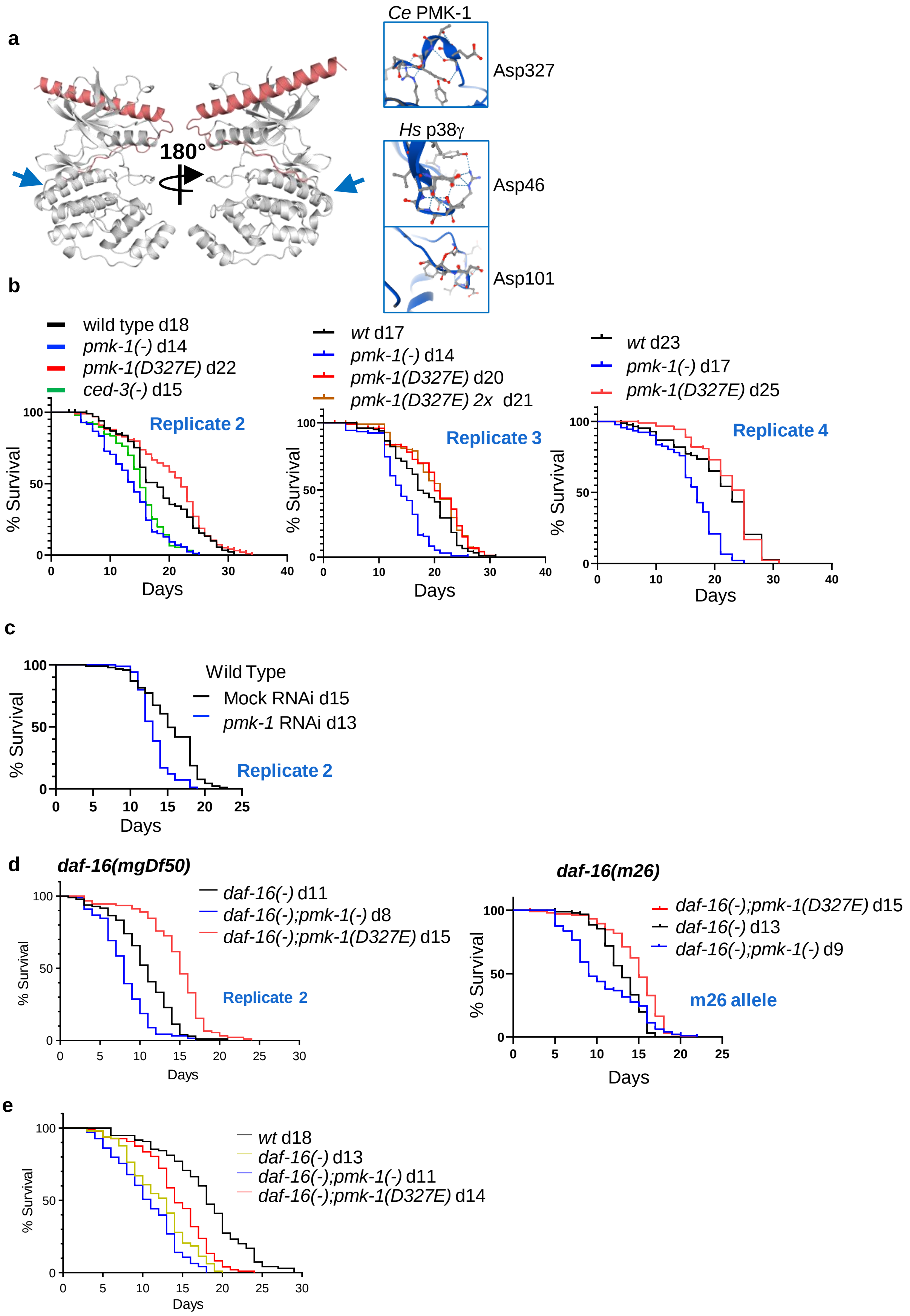

Supplementary Figure 1. Aging assays for caspase cleavage resistant PMK-1 mutants.

a Alphafold model of PMK-1. Arrows indicate cleavage site. Aspartate residue at cleavage site shown. **b** Replicates of aging assays for *pmk-1* null and cleavage resistant *pmk-1(D327E)* mutants. **c** Replicates of aging assay for *pmk-1* RNAi. **d** Replicates of effects of *pmk-1* null and *pmk-1(D327E)* mutations on *daf-16* mutant aging. **e** Aging assay evaluating restoration of *daf-16(-)* life span by cleavage resistant *pmk-1(D327E)* mutation. All aging replicates and statistics are shown in **Supplementary Data 1**.

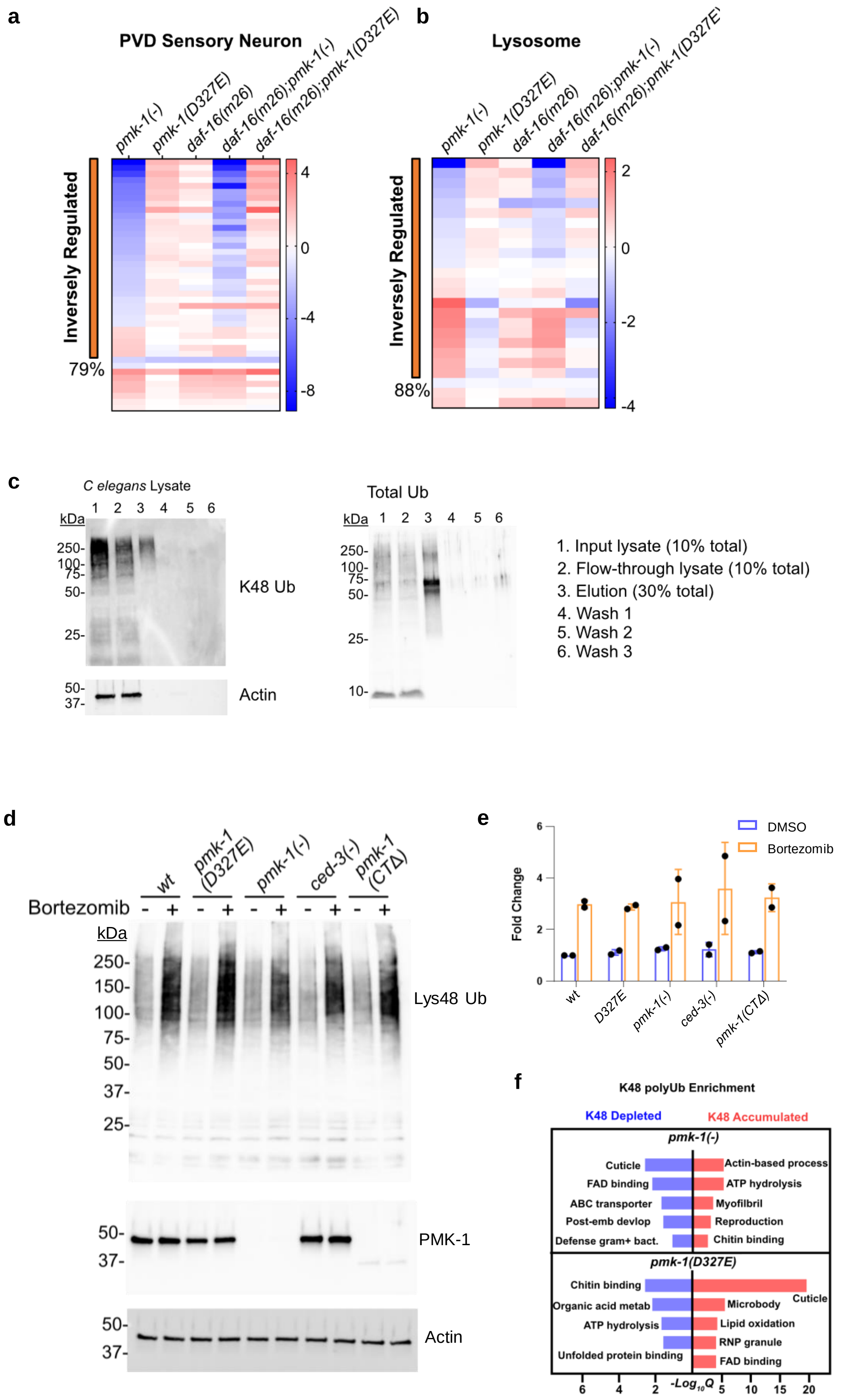
a
PVD Sensory Neuron
pmk-1(-)
pmk-1(D327E)
daf-16(m26)
daf-16(m26);pmk-1(-)
daf-16(m26);pmk-1(D327E)
Inversely Regulated
79%
4
0
-4
-8
b
Lysosome
pmk-1(-)
pmk-1(D327E)
daf-16(m26)
daf-16(m26);pmk-1(-)
daf-16(m26);pmk-1(D327E)
Inversely Regulated
88%
2
0
-2
-4
c
C elegans Lysate
1 2 3 4 5 6
kDa
250-
100-
75-
50-
25-
K48 Ub
50-
37-
Actin
Total Ub
kDa
1 2 3 4 5 6
250-
100-
75-
50-
25-
10-
1. Input lysate (10% total)
2. Flow-through lysate (10% total)
3. Elution (30% total)
4. Wash 1
5. Wash 2
6. Wash 3
d
wt
pmk-1 (D327E)
pmk-1(-)
ced-3(-)
pmk-1 (CTΔ)
Bortezomib - + - + - + - + - +
kDa
250-
150-
100-
75-
50-
37-
25-
Lys48 Ub
50-
37-
PMK-1
50-
37-
Actin
e
DMSO
Bortezomib
Fold Change
6
4
2
0
wt
D327E
pmk-1(-)
ced-3(-)
pmk-1(CTΔ)
f
K48 polyUb Enrichment
K48 Depleted
K48 Accumulated
pmk-1(-)
Cuticle
FAD binding
ABC transporter
Post-emb devlop
Defense gram+ bact.
Actin-based process
ATP hydrolysis
Myofilbril
Reproduction
Chitin binding
pmk-1(D327E)
Chitin binding
Organic acid metab
ATP hydrolysis
Unfolded protein binding
Cuticle
Microbody
Lipid oxidation
RNP granule
FAD binding
6 4 2 -Log10Q 5 10 15 20

Supplementary Figure 2. *pmk-1*-regulated sensory neuron and lysosome genes for *daf-16(m26)* allele dependency and global K48-specific Ub linkage enrichments with Bortezomib treatment.

a-b Sensory neuron and lysosome genes heatmap for *daf-16(m26)* allele. *daf-16(mgDf50)* allele was shown in **Fig.2**. PMK-1-dependent genes clustered by inverse regulation with PMK-1(D327E) and plotted with Log2-fold change compared to wild type. n=2 biological replicates. **c** Western blot of K48 Ub and total Ub showing the polyubiquitinated protein enrichment for mass spectrometry analysis. Data from one experiment with hundreds of worms per sample. **d-e** Western blots and quantification of bortezomib treatment induced K48 linkages to a comparable extent in *pmk-1(-)* null and *pmk-1(D327E)* cleavage resistant mutants. n=2 biological replicates. Data are presented as mean values +/- SD**. f** Pathway enrichment of K48 Ub antibody associated proteins that are inversely regulated by *pmk-1(-)* and *pmk-1(D327E).* n=2 biological replicates.

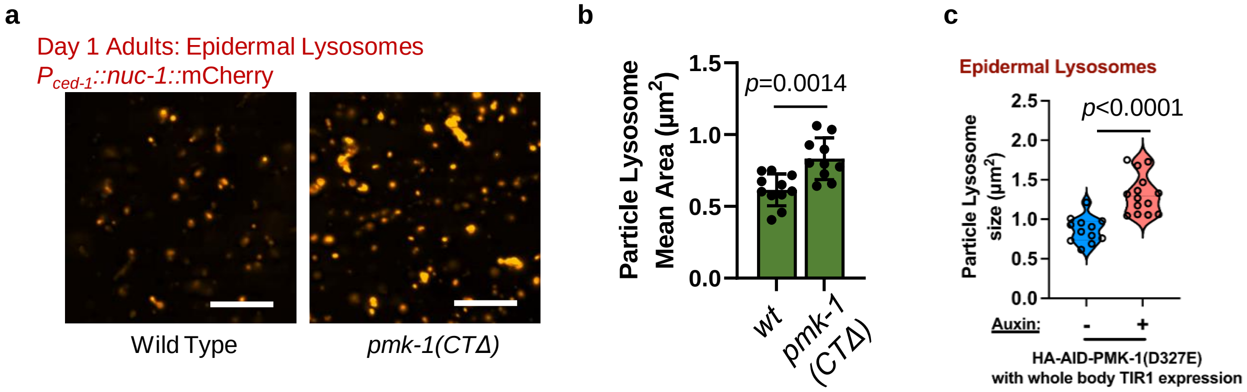

Supplementary Figure 3. Lysosome phenotype of *pmk-1(CTΔ)* and auxin induced degradation of PMK-1(D327E).

a-b Day 1 adult lysosome morphology and quantitation of *pmk-1(CTΔ)* mutants compared to wild-type. Scale bar 10μm. n=10 animals. Data are presented as mean values +/- SD. *p* value from two-tailed unpaired t test with Welch's correction. **c** Auxin-induced degradation of PMK-1(D327E) is similar to *pmk-1* null with increased lysosome particle size. *p* value from two-tailed unpaired t test with Welch's correction.

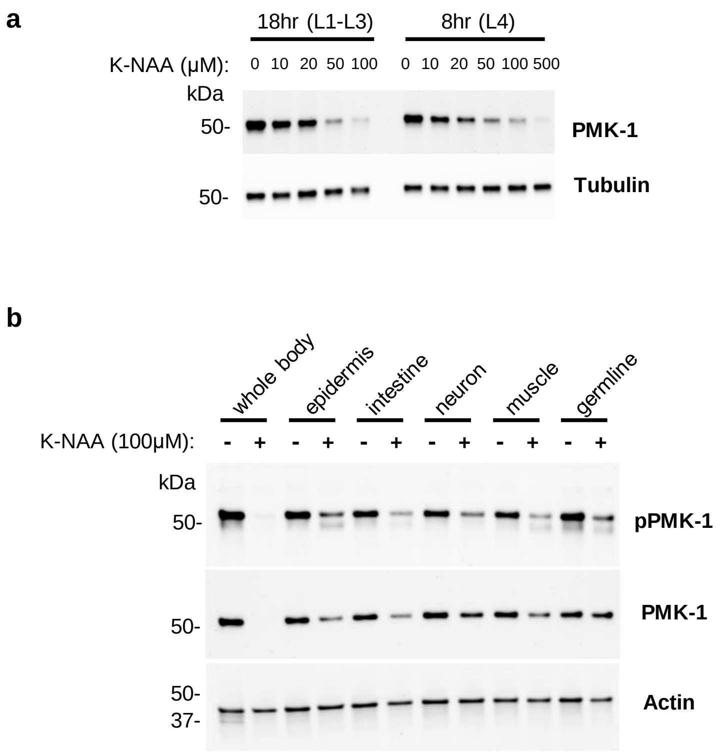

Tissue	Promoter
whole body	*eft-3*
epidermis	*col-10*
intestine	*ges-1*
neuron	*rgef-1*
muscle	*myo-3*
germline	*sun-1*

Supplementary Figure 4. Dose-dependent and tissue-specific auxin-induced degradation of PMK-1 Protein.

a Western blot showing diminishing PMK-1 protein with increasing K-NAA concentration. Both early developmental and late developmental to young adult stage were tested. 100µM K-NAA was used for all auxin induced degradation in this study. Animals were treated from hatching to young adult continuously unless indicated otherwise. **b** Tissue specific auxin-induced degradation of PMK-1. Tissue specific promoters used to express TIR1 E3 ligase are listed in the figure. Animals were treated from hatching to young adult continuously.

a

	UNC-62	FOS-1	JUN-1
**Pro-Directed Sites**	79-85: PTSTPMM<br>119-125: ATSTPRD<br>223-229: PPMSPGS<br>235-241: HSSSPSM<br>234-249: GGATPMH<br>324-330: AVSSPST<br>338-344: QDSTPLS<br>344-350: SGETPMG<br>478-484: SEQSPGP<br>482-488: PGPSPDS<br>494-500: ANYSPDP	57-63: TDNSPLT<br>60-66: SPLTPCT<br>63-69: TPCTPYY<br>91-97: DIPSPLT<br>94-100: SPLTPNI<br>99-105: NISSPLT<br>102-108: SPLTPHP<br>132-138: AASSPMV<br>284-290: EQHSPVE<br>396-402: PITTPSR<br>411-417: QNQTPQS<br>437-443: TGLTPSG<br>449-455: FVSTPTP<br>451-457: STPTPIQ	14-20: SSESPEV<br>79-85: AVSSPVL<br>91-97: AAFSPIT<br>94-100: SPITPAS<br>112-118: LGNTPIT<br>125-131: PLSSPTL<br>161-167: SSNSPDS<br>201-207: LNLTPPQ
**Docking**	205-212: KGKMPLDI<br>460-469: RAGRSGQMNV	359-366: RKIPKIEL	36-45: KKDRRQDMEV

b

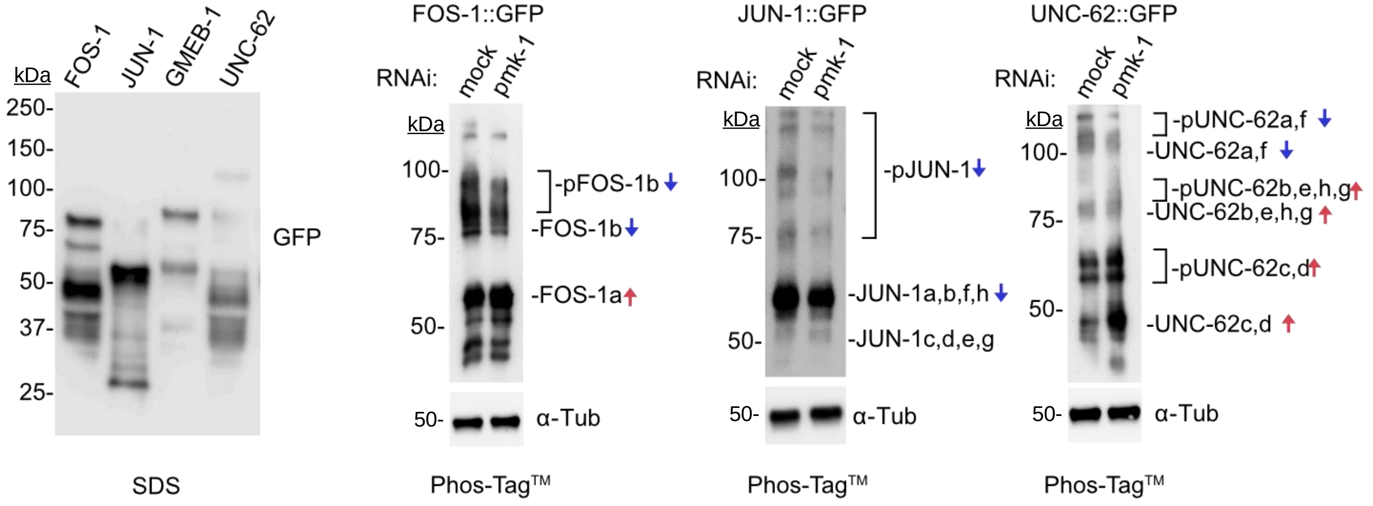

Supplementary Figure 5. FOS-1 JUN-1 and UNC-62 phosphorylation with *pmk-1* RNAi.

a Motif analyses for putative phospho-sites and MAPK docking based on eukaryotic linear motif analysis (Eukaryotic Linear Motif database). **b** Western blots of SDS and Phos-Tag™ gel of GFP tagged FOS-1, JUN-1 and UNC-62. Isoforms were estimated based on molecular weight. Phosphorylated form was determined by comparison of Phos-Tag™ and SDS. Red arrow indicates *pmk-1* RNAi treatment increased accumulation. Blue arrow indicates *pmk-1* RNAi treatment decreased accumulation. Data from one experiment with hundreds of worms per sample.

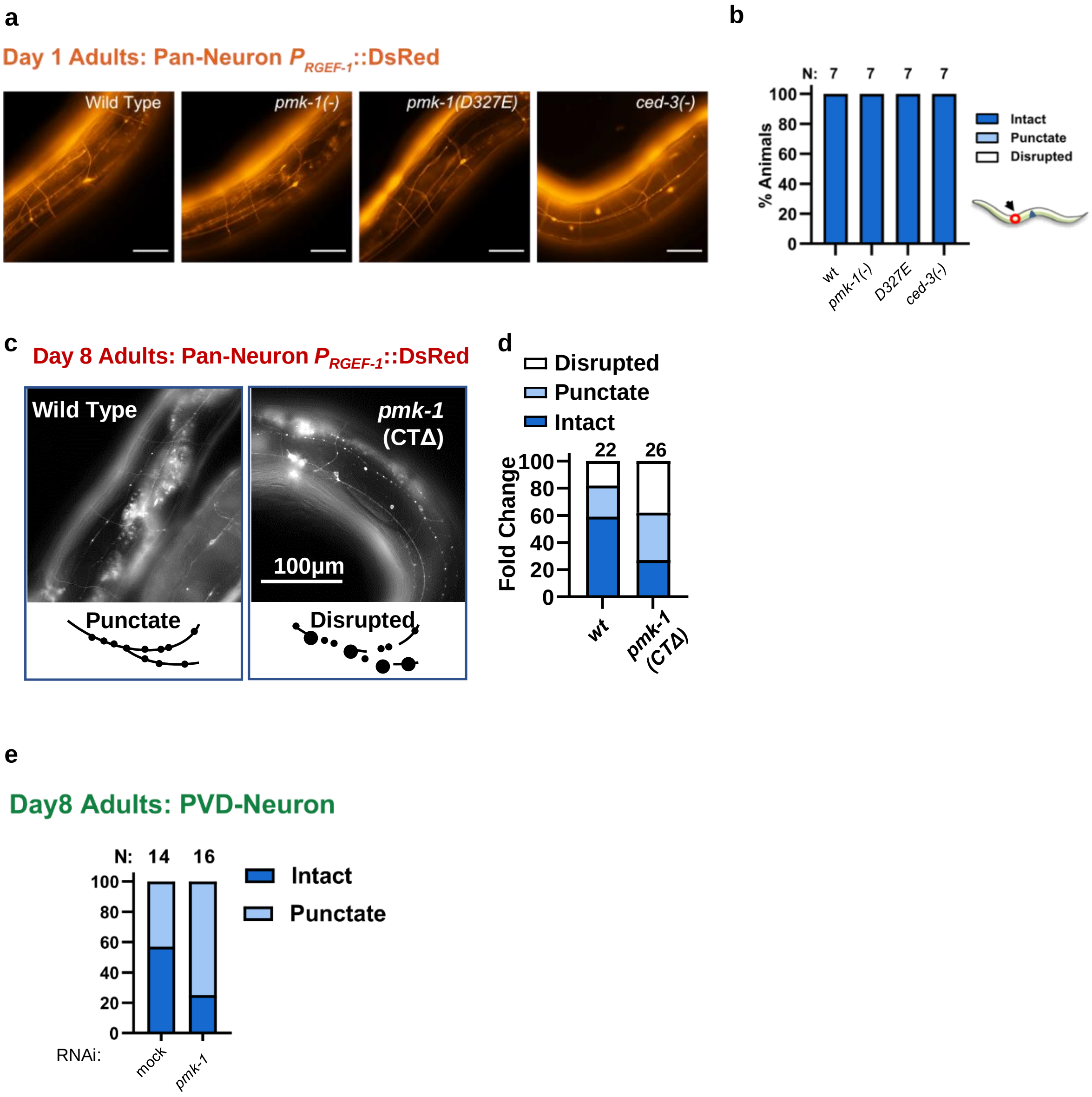

Supplementary Figure 6. Functions of *pmk-1* in neuronal integrity with aging.

a-b Day 1 images and quantification of large mid-body neuronal morphology. N, number of animals.**c-d** Day 8 images and quantification of large mid-body neuronal morphologies in *pmk-1(CTΔ)* mutants. **e** Quantification of Day 8 PVD neuronal morphologies with *pmk-1* RNAi. Number of animals listed on top of each column.

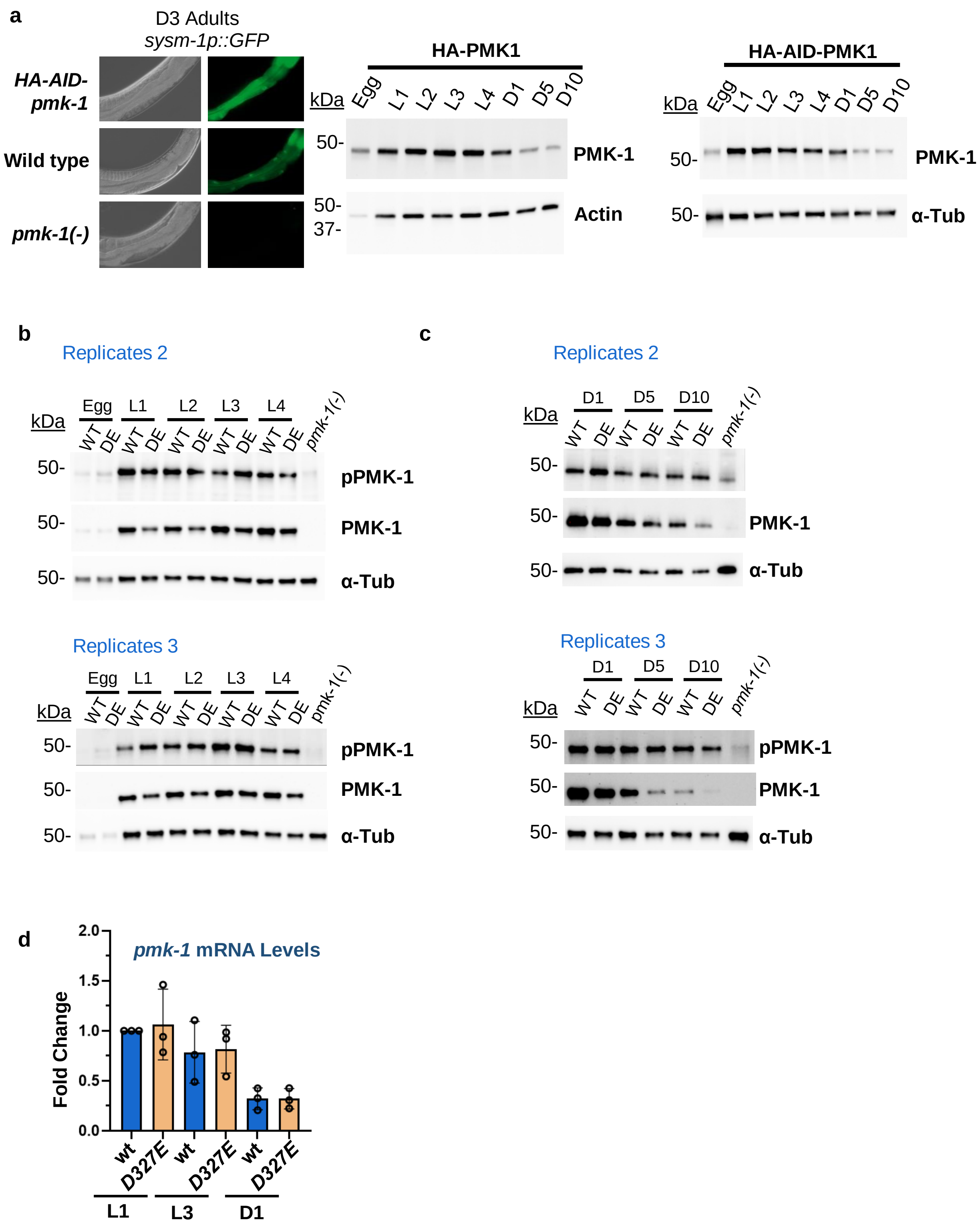

a
D3 Adults
sysm-1p::GFP
HA-AID-pmk-1
Wild type
pmk-1(-)
HA-PMK1
kDa
Egg L1 L2 L3 L4 D1 D5 D10
50-
PMK-1
50-
37-
Actin
HA-AID-PMK1
kDa
Egg L1 L2 L3 L4 D1 D5 D10
50-
PMK-1
50-
α-Tub
b
Replicates 2
kDa
Egg L1 L2 L3 L4
WT DE WT DE WT DE WT DE WT DE pmk-1(-)
50-
pPMK-1
50-
PMK-1
50-
α-Tub
Replicates 3
kDa
Egg L1 L2 L3 L4
WT DE WT DE WT DE WT DE WT DE pmk-1(-)
50-
pPMK-1
50-
PMK-1
50-
α-Tub
c
Replicates 2
kDa
D1 D5 D10
WT DE WT DE WT DE pmk-1(-)
50-
50-
PMK-1
50-
α-Tub
Replicates 3
kDa
D1 D5 D10
WT DE WT DE WT DE pmk-1(-)
50-
pPMK-1
50-
PMK-1
50-
α-Tub
d
pmk-1 mRNA Levels
Fold Change
2.0
1.5
1.0
0.5
0.0
wt D327E wt D327E wt D327E
L1 L3 D1

Supplementary Figure 7. Validation of endogenous PMK-1 tag and replicates of PMK-1 expression with development and aging.

a PMK-1-dependent reporter gene induced with HA-AID-tagged PMK-1 at day3 adulthood. HA-PMK-1 and HA-AID-PMK-1 expression in development and aging. Data from one experiment with hundreds of worms per sample. **b-c** Western blot replicates for N-terminal HA tagged PMK-1 in development and aging. **d** Quantitative RT-PCR of *pmk-1* (wt) versus *pmk-1(D327E)* mRNAs normalized to ama-1. Data are presented as mean values +/- SD. Each open circle represents one biological replicate. n=3 biological replicates.

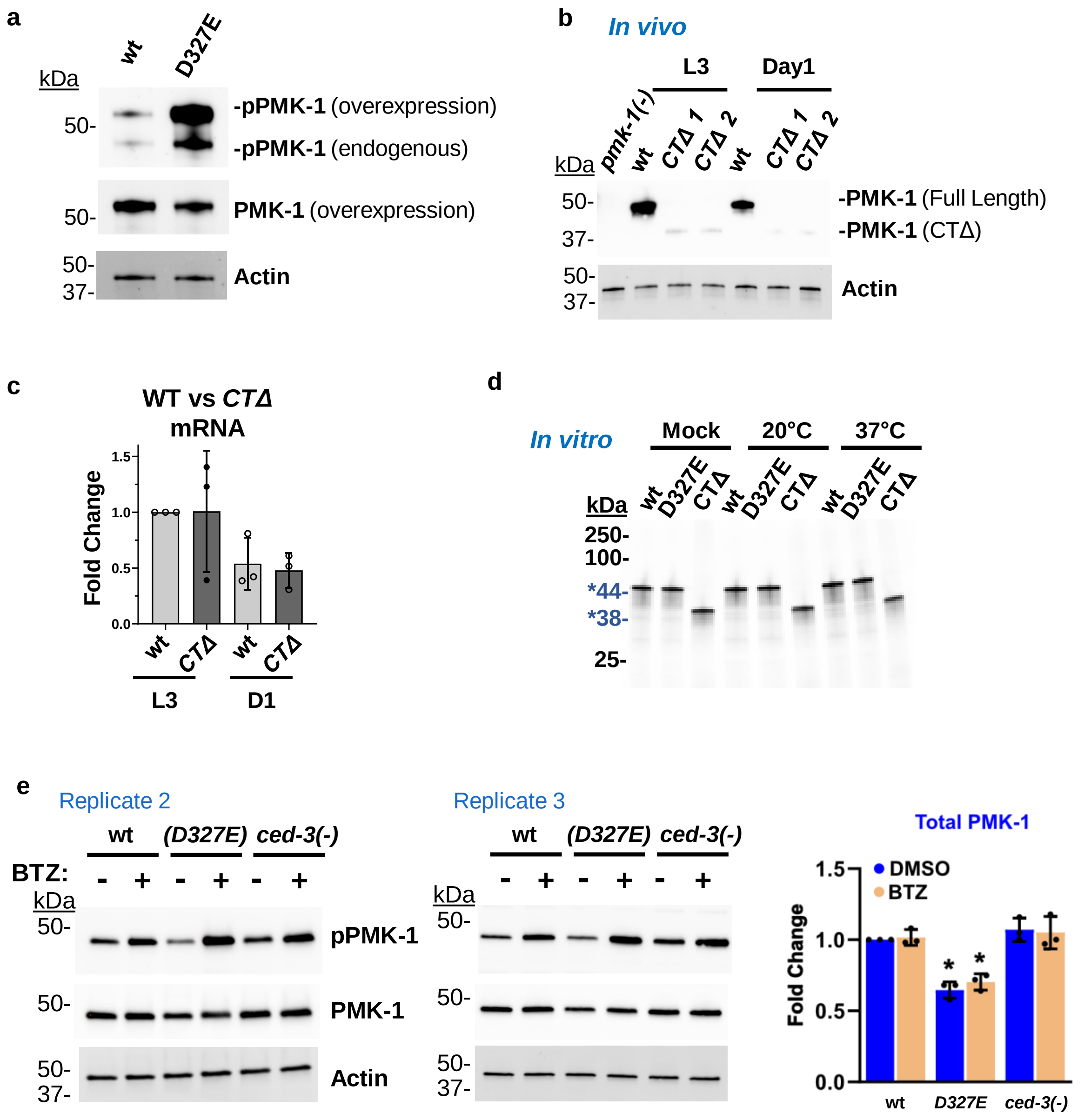

Supplementary Figure 8. PMK-1 overexpression, pPMK-1 expression in *ced-3(-)* mutant animals and expression of *pmk-1(CTΔ)* mutant.

a Replicate of PMK-1 overexpression in **Fig.5f**. **b** *In vivo* expression of PMK-1(CTΔ). **c** Quantitative RT-PCR of *pmk-1* (wt) versus *pmk-1(CTΔ)* mRNAs normalized to ama-1. Data shown as mean values +/- SD. n=3 biological replicates. **d** *In vitro* expression of CTΔ mutation. Data from one experiment. **e** Replicate of Bortezomib treatment in **Fig.5l** and quantification of total PMK-1 levels in 3 biological replicates. Data are presented as mean values +/- SD.

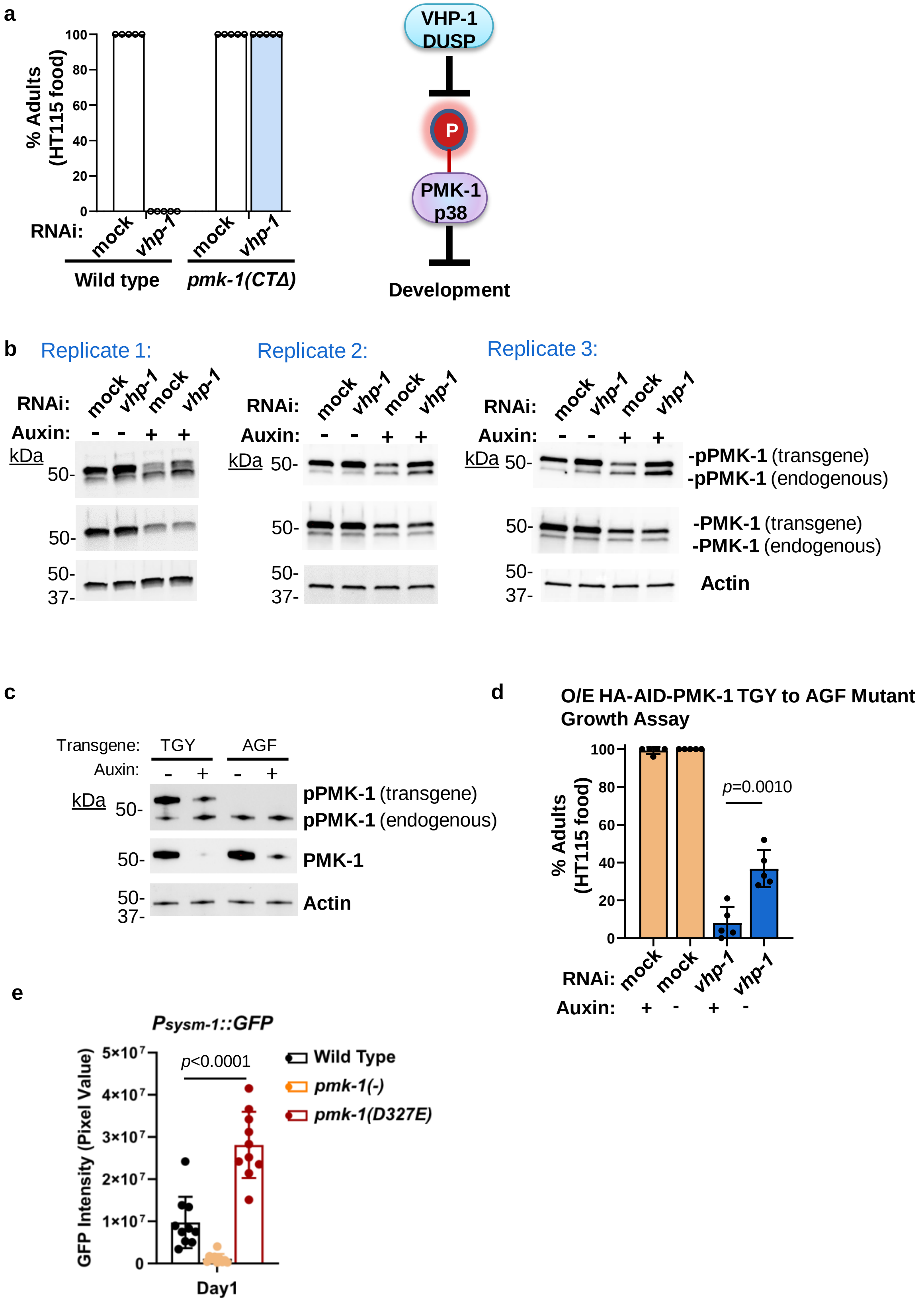
a
% Adults
(HT115 food)
100
80
60
40
20
0
RNAi:
mock
vhp-1
mock
vhp-1
Wild type
pmk-1(CTΔ)
VHP-1
DUSP
P
PMK-1
p38
Development
b
Replicate 1:
Replicate 2:
Replicate 3:
RNAi:
mock
vhp-1
mock
vhp-1
Auxin:
- - + +
kDa
50-
37-
-pPMK-1 (transgene)
-pPMK-1 (endogenous)
-PMK-1 (transgene)
-PMK-1 (endogenous)
Actin
c
Transgene:
TGY
AGF
Auxin:
- + - +
kDa
50-
37-
pPMK-1 (transgene)
pPMK-1 (endogenous)
PMK-1
Actin
d
O/E HA-AID-PMK-1 TGY to AGF Mutant
Growth Assay
% Adults
(HT115 food)
100
80
60
40
20
0
p=0.0010
RNAi:
mock
mock
vhp-1
vhp-1
Auxin:
+ - + -
e
Psysm-1::GFP
GFP Intensity (Pixel Value)
5×10^7
4×10^7
3×10^7
2×10^7
1×10^7
0
p<0.0001
Day1
Wild Type
pmk-1(-)
pmk-1(D327E)

Supplementary Figure 9. *pmk-1*-dependent growth stall, replicates for Western blot, and transgene of pmk-1 TGY to AGF mutant rescues growth on *vhp-1* RNAi.

a Developmental delay from *vhp-1* RNAi is *pmk-1*-dependent as shown by alleviation of the stall by loss of pmk-1 function. n=5 biological replicates with 20 animals per replicate. Data are presented as mean values +/- SD. **b** Western blot for conditions showing pPMK-1 and PMK-1 changes with vhp-1 RNAi and auxin analog (K-NAA) treatments. **c** Western blot of pPMK-1 of either wild-type PMK-1 sequence (TGY) or phospho-dead (AGF) mutation showing AGF mutation eliminates phosphorylation. Data from one experiment. **d** Developmental rate assay for pmk-1 TGY to AGF transgene mutant. Animals were treated with mock or vhp-1 RNAi with (+) or without (-) auxin to allow transgene expression. Each dot corresponds to a plate with 25 to 50 animals. n=5 biological replicates. Data are presented as mean values +/- SD. *p* value from two-tailed unpaired t-test. **e** Quantification of $P_{sysm\text{-}1}$GFP on Day 1 of aging. n=10 animals. Data are presented as mean values +/- SD. *p* value from two-tailed, unpaired t-test.
