# Supplementary material for: Modulating p38 MAPK signaling by proteostasis mechanisms supports tissue integrity during growth and aging
Source: Nat Commun. 2023 Jul 28;14:4543. doi: 10.1038/s41467-023-40317-7 (PMC10382525; doi:10.1038/s41467-023-40317-7)
Supplement: Supplementary file 3 — Description of Additional Supplementary Files [file 41467_2023_40317_MOESM3_ESM.pdf]

Description of Additional Supplementary Files

File Name: Supplementary Data 1
Description: Statistics of Aging Assays.

File Name: Supplementary Data 2
Description: pmk-1 Dependent Genes mRNA seq.

File Name: Supplementary Data 3
Description: Transcription Factor Enrichment.

File Name: Supplementary Data 4
Description: Polyubiquitin Enrichment.

File Name: Supplementary Data 5
Description: *C. elegans* strains used in this study.

File Name: Supplementary Data 6
Description: Primers used for RT_qPCR.
